# Mechanistic analysis of multi-omics datasets to generate kinetic parameters for constraint-based metabolic models

**DOI:** 10.1186/1471-2105-14-32

**Published:** 2013-01-30

**Authors:** Cameron Cotten, Jennifer L Reed

**Affiliations:** 1 Department of Chemical and Biological Engineering, University of Wisconsin-Madison, 1415 Engineering Dr, Madison, WI 53706, USA; 2 DOE Great Lakes Bioenergy Research Center, University of Wisconsin-Madison, 1415 Engineering Dr, Madison, WI 53706, USA

**Keywords:** Metabolic engineering, Kinetics, Central metabolism, Constraint-based, FBA

## Abstract

**Background:**

Constraint-based modeling uses mass balances, flux capacity, and reaction directionality constraints to predict fluxes through metabolism. Although transcriptional regulation and thermodynamic constraints have been integrated into constraint-based modeling, kinetic rate laws have not been extensively used.

**Results:**

In this study, an *in vivo* kinetic parameter estimation problem was formulated and solved using multi-omic data sets for *Escherichia coli*. To narrow the confidence intervals for kinetic parameters, a series of kinetic model simplifications were made, resulting in fewer kinetic parameters than the full kinetic model. These new parameter values are able to account for flux and concentration data from 20 different experimental conditions used in our training dataset. Concentration estimates from the simplified kinetic model were within one standard deviation for 92.7% of the 790 experimental measurements in the training set. Gibbs free energy changes of reaction were calculated to identify reactions that were often operating close to or far from equilibrium. In addition, enzymes whose activities were positively or negatively influenced by metabolite concentrations were also identified. The kinetic model was then used to calculate the maximum and minimum possible flux values for individual reactions from independent metabolite and enzyme concentration data that were not used to estimate parameter values. Incorporating these kinetically-derived flux limits into the constraint-based metabolic model improved predictions for uptake and secretion rates and intracellular fluxes in constraint-based models of central metabolism.

**Conclusions:**

This study has produced a method for *in vivo* kinetic parameter estimation and identified strategies and outcomes of kinetic model simplification. We also have illustrated how kinetic constraints can be used to improve constraint-based model predictions for intracellular fluxes and biomass yield and identify potential metabolic limitations through the integrated analysis of multi-omics datasets.

## Background

Constraint-based models consider the physico-chemical constraints that exist on metabolism, including mass balances, flux capacities, thermodynamics, and transcriptional regulation of metabolic enzymes [1,2]. One common constraint-based modeling approach, flux balance analysis (FBA) uses mass balance and reaction reversibility constraints to predict metabolic fluxes that maximize flux through a reaction or combination of reactions [2]. FBA typically is used to find flux distributions that maximize biomass production or ATP generation per total flux [3]. Some constraint-based models add transcriptional regulatory constraints to further limit flux values [4-6] when the associated enzymes are known to be unexpressed in certain conditions. Thermodynamic constraints have also been imposed to limit the direction a given reaction can proceed, and thermodynamic metabolic flux analysis (TMFA) uses these constraints to ensure that reactions only proceed in the thermodynamically-favorable direction [7-10]. TMFA models were some of the first constraint-based models that directly accounted for intracellular metabolite concentrations.

Constraint-based models and kinetic differential equation models have largely been divergent methodologies in systems biology. In constraint-based modeling, omission of kinetic considerations is generally seen as an advantage of the methodology, since determining kinetic information for an entire metabolic network is currently infeasible. However, FBA and TMFA predictions are not always consistent with experimental observations because of kinetic limitations on native enzymes. To account for these kinetic limitations, there have been some efforts to integrate kinetic expressions into constraint-based models. Beg *et al.*[11] were able to improve predictions of cellular growth rates by constraining fluxes using individual enzyme activity, enzyme volume needed to achieve a given flux, and the total enzyme volume. More recently, Yizhak *et al.*[12] integrated metabolomic and proteomic data into Michaelis-Menten kinetic expressions using *in vitro K*_*m*_ parameters to more accurately predict flux changes (increases/decreases) than methods that did not consider kinetics. Similarly, dynamic flux balance analysis models have also used Michaelis-Menten kinetic expressions to constrain substrate uptake rates based on reactor concentrations [13-15].

In the present study, we estimated kinetic parameters in a kinetic model of central *Escherichia coli* metabolism by integrating fluxomic, proteomic, and metabolomics data. Data published by Ishii *et al.*[16] from single deletion mutants and the parental strain (at different growth rates) were used to construct a weighted sum of least squares (WSLS) parameter estimation problem using kinetic rate laws. Initially rate laws reported by Chassagnole *et al.*[17] were used; however, these rate laws resulted in parameters with large confidence intervals. A simplified kinetic model was subsequently constructed that resulted in smaller confidence intervals for kinetic parameters. Predictions from the kinetic model for metabolite concentrations and kinetic parameters were used to draw conclusions about metabolite-mediated inhibition and activation effects on enzymes, and distance from equilibrium for reactions in central metabolism. Using independent data sets, we then used the kinetic model to predict flux ranges that are consistent with estimated kinetic parameters and concentration data. We subsequently incorporated these flux ranges as constraints into a constraint-based model to improve predictions over FBA. This new constraint-based model with kinetic constraints is one of the most detailed constraint-based models with kinetic constraints to date, and is one of a few to only use *in vivo* estimates of kinetic parameters.

## Methods

### Constraint-based metabolic model

A central *E. coli* constraint-based metabolic model previously reported by Palsson [18] was used that included glycolysis, pentose phosphate, oxidative phosphorylation, and the citric acid pathways. This model was chosen because it includes the fluxes, proteins, and metabolites measured by Ishii *et al.*[16]. This model included mass balance constraints for central metabolic intermediates, as well as energy and redox carriers. Fluxes from Ishii *et al.*[16] were adjusted slightly to satisfy mass balance constraints and energy requirements in the metabolic models by minimizing the Euclidean distance between the fluxes in the constraint-based model and the reported flux distributions. These adjusted flux measurements were then used to estimate kinetic parameters and evaluate model-predicted fluxes from a constraint-based model with kinetic constraints. The most recent genome-scale model of *E. coli* (iJO1366) [19] was also used to evaluate the effects of kinetic constraints. A few reactions were excluded from the model (EDD, HEX1, F6PA, FBA3, FLDR2, and DRPA) so that the adjusted fluxes more closely matched the measured flux patterns.

### Kinetic parameter estimation using multi-omic data

In general, kinetic rate laws can be written in the form *v=e f(c;*θ*)*, where the flux (*v*) is proportional to the concentration of the associated enzyme (*e*) and some mechanistic function (*f*) of metabolite concentrations (*c*) and kinetic parameters (θ*)*. To estimate kinetic parameters, data on fluxes, metabolite, and enzyme concentrations generated by Ishii *et al.*[16] was used. Rate laws based on those reported by Chassagnole *et al.*[17] were used (Additional file 1: Table S1). The adenylate energy charge (Equation 1c) was also constrained to be at least 0.8, based on typical experimental values [20]. This constraint was used since ATP concentrations were not measured, but were used in many kinetic rate laws. The adenylate energy charge then relates ATP concentration to measured AMP and ADP concentrations. Parameters in the kinetic model were then estimated using nonlinear regression. The adjusted flux measurements for each experimental condition were used in a kinetic model, where a weighted sum of least squares (WSLS) kinetic parameter estimation problem was formulated (Equations 1a-1e). In this case, fluxes and kinetic constraints were used to find kinetic parameters that were most consistent with measured metabolite and protein concentrations. The full WSLS parameter estimation problem is:

(1a) $$\min_{c,e,\mathrm{AEC},\theta }\;\sum_{l}\left[\sum_{i}W_{\mathrm{il}}^{c}{\left(c_{\mathrm{il}}-c_{\mathrm{il}}^{\exp}\right)}^{2}+\sum_{k}W_{\mathrm{kl}}^{e}{\left(e_{\mathrm{kl}}-e_{\mathrm{kl}}^{\exp}\right)}^{2}\right]$$

such that

(1b) $$v_{\mathrm{kl}}=e_{\mathrm{kl}}f_{k}\left(c_{l},\theta \right)\forall l\in L,k\in K$$

(1c) $$\left(c_{\mathrm{ATP},l}+0.5\cdot c_{\mathrm{ADP},l}\right)/\left(c_{\mathrm{ATP},l}+c_{\mathrm{ADP},l}+c_{\mathrm{AMP},l}\right)\geq 0.8\;\forall l\in L$$

(1d) $$0.001\leq e_{\mathrm{kl}},c_{\mathrm{il}}\leq 10\;\forall l\in L,k\in K,i\in I$$

(1e) $$0.35K_{\mathrm{eq},k}^{o}\leq K_{\mathrm{eq},k}\leq 2.85K_{\mathrm{eq},k}^{o}\forall k\in K$$

Here, *I* is the set of metabolites, *K* is the set of reactions with kinetic rate laws, *L* is the set of experimental conditions, and θ is a vector of all kinetic parameter values. Parameters except for equilibrium constants were assigned global upper and lower bounds of 10^4^ and 10^-6^, respectively (in their respective units). The estimates of metabolite and enzyme concentrations (*c*_*il*_ and *e*_*kl*_) are variables and are adjusted to be as close as possible to the experimentally-measured metabolite and enzyme concentrations (*c*_*il*_^exp^ and *e*_*kl*_^exp^). Fluxes with kinetic rate laws (*v*_*kl*_) were fixed to the adjusted values from the constraint-based model. Biologically-relevant global upper and lower bounds were enforced on metabolite concentrations (0.001 mM and 10 mM) and enzyme concentrations (0.001 mg protein/gDCW and 10 mg protein/gDCW). Kinetic parameters were allowed to vary freely, except for equilibrium constants that were required to be within 35% to 285% of their *in vitro* measured values in standard conditions [21], corresponding to changes of 2.6 kJ/mol in the Gibbs free energy change of reaction. The weights in the WSLS objective function, *W*_*il*_^*c*^ and *W*_*kl*_^*e*^, were assigned as the inverse of experimental variances for the metabolic and enzyme concentration measurements, respectively. This weighting method was chosen to keep residuals on the same scale and assign less importance to concentrations with measurements that were more uncertain. Experimental sample variances were calculated from concentration measurements from 4 biological replicates of the parental strain growing steadily at 0.2 h^-1^.

The WSLS parameter estimation problem was solved using CONOPT3 as called by GAMS 23.3 (GAMS Development Corporation, Washington, DC) from multiple starting points to explore the non-convex feasible space and find multiple local optimal solutions. The feasible solution with the lowest objective value was selected as the set of optimal kinetic parameters values and concentrations. Confidence intervals for the set of kinetic parameters were estimated by determining the sensitivity of enzyme and metabolite concentrations to parameter values. This sensitivity was determined by making small perturbations to parameter values one at a time and re-optimizing Equations 1a-1e. These sensitivities were used to determine confidence intervals in a method described by Antoniewicz *et al.*[22]. Generally, smaller WSLS values and higher sensitivities lead to smaller confidence intervals and more confidence in parameters. Five conditions (Δ*pgm*, Δ*gpmA*, Δ*zwf*, Δ*tktB*, and parental strain at *D*=0.5 h^-1^) were randomly drawn from the set of experimental conditions and left out during parameter estimation, to form an independent test set to later evaluate the resulting constraint-based model.

### Predictions using FBA with kinetic constraints (KFBA)

A method to improve FBA using kinetic constraints was developed. This method combines a constraint-based model with flux ranges determined by a kinetic model. Upper and lower kinetic flux bounds for intracellular fluxes were estimated using the kinetic model for each test condition using the rate laws, kinetic parameter confidence intervals, and measured metabolite and enzyme concentrations. Minimum (*v*_*k*_^min^) and maximum (*v*_*k*_^max^) possible values for each flux were determined by fixing the concentration measurements and allowing kinetic parameters to vary within their 95% confidence intervals. Metabolite and enzyme concentration measurements were from experimental conditions that were not used during kinetic model parameter estimation. Because a steady-state metabolic flux distribution could not be found in the constraint-based model that was consistent with all the flux bounds calculated by the kinetic model, a solution was found with the minimum number of kinetic bounds violated (n*). For the five test sets evaluated using the central metabolic model, a median of 16 out of 22 kinetic bounds could be enforced. The maximum number of kinetic bounds that could be violated was set to n* for each condition in our kinetic FBA problem

(2a) $$(\text{KFBA}):\;\min_{v,\;y^{+},\;y^{-}}\;v_{\mathrm{PTS}}$$

such that

(2b) S·v=0

(2c) $$LB_{j}\leq v_{j}\leq UB_{j}\;\forall j\in J$$

(2d) $$\begin{array}{l} \left(LB_{k}-v_{k}^{\min}\right)y_{k}^{-}+v_{k}^{\min}\leq v_{k}\leq \left(UB_{k}-v_{k}^{\max}\right)y_{k}^{+}\\ \;+v_{k}^{\max}\;\forall k\in K \end{array}$$

(2e) $$\sum_{k}y_{k}^{+}+y_{k}^{-}\leq n^{*}$$

Here, *J* is the set of all reactions, *K* is the subset of reactions with kinetic rate laws, and the binary variables *y*^*+*^ and *y*^*-*^ indicate whether an upper or lower kinetic bound is violated. A general lower bound (*LB*=0 or −1000 mmol/gDW/h for irreversible and reversible reactions, respectively) and an upper bound (*UB*=1000 mmol/gDW/h) were used for all reactions, including those with kinetic rate laws. All fermentative pathways were blocked (i.e., *LB* and *UB* set to 0), as only carbon dioxide was produced in experiments. The choice for *UB* and *LB* did not affect predictions, as fluxes did not go to these limits at the optimal solutions and the kinetic bounds were between the *LB* and *UB* values. The cellular growth rates (*v*_biomass_) were fixed to the chemostat dilution rate. An analogous FBA problem was formulated using Equations 1a-2c. Since the FBA and KFBA solutions are not necessarily unique, we selected the alternate optimal solution with the lowest sum of squared flux values [23] by solving an additional minimization problem, which gives a unique answer. Residuals were calculated for the subset of measured fluxes as the squared difference between the adjusted flux measurement and FBA or KFBA flux prediction. When calculating the mean residual, only one flux residual was used for fluxes that must be directly proportional to one another based on the network stoichiometry (e.g., reactions in a linear pathway).

## Results

**Figure 1:**
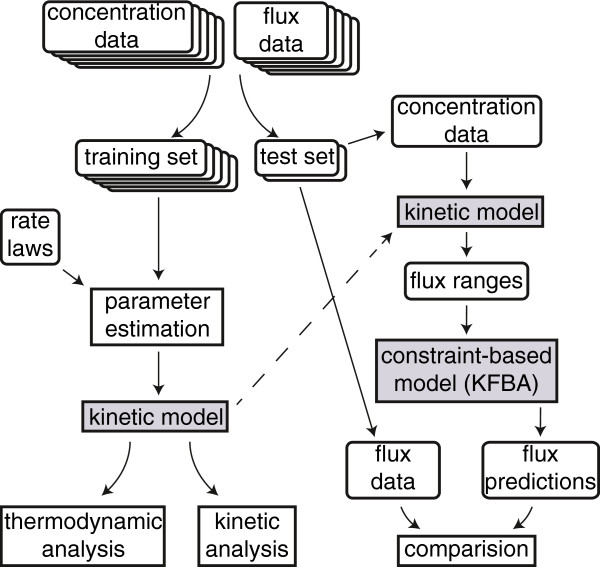
**Methods summary.** Kinetic rate laws were fit to fluxomic, proteomic, and metabolomics data using nonlinear least squares regression. Of the 25 experimental conditions that were available, 20 were used for fitting, and 5 were used as a test set. Concentration estimates from parameter fitting were used to examine the thermodynamic and kinetic limitations of the central metabolism. The fit kinetic parameters were used to add kinetic constraints to a constraint-based model to estimate internal fluxes and biomass yields. These estimates were compared to experimental data.

In this study, we developed a kinetic and thermodynamic approach to integrate multi-omics datasets to constrain metabolic fluxes (Figure 1). A kinetic model with rate laws for a subset of enzymes in glycolysis and pentose phosphate pathway was developed [17]. Using fluxomic, proteomic, and metabolomic data, *in vivo* kinetic parameters were estimated by fitting the kinetic model to metabolite and protein concentration measurements. A simplified kinetic model with an equivalent fit to the experimental data, but with fewer kinetic parameters, was subsequently generated. The best estimates for metabolite concentrations and kinetic parameter values from the parameter estimation problem in the simplified kinetic model were used to draw conclusions about kinetic and thermodynamic control in central metabolism. The simplified kinetic model was also used to generate tighter flux bounds for constraint-based models, allowing us to evaluate predictions for experimental conditions that were not used in train the kinetic model.

### *In vivo* kinetic parameter estimation

**Figure 2:**
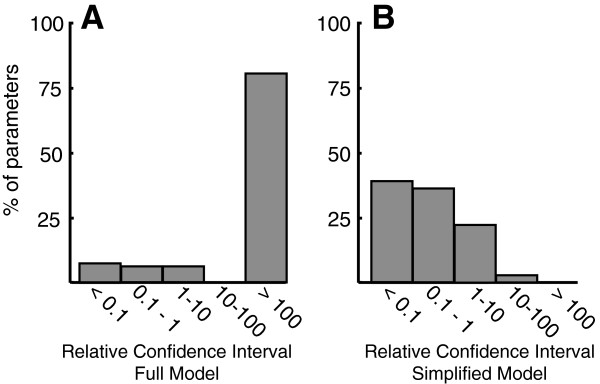
**Histogram of Relative Confidence Intervals.** Relative confidence intervals in the full (**A**) and simplified model (**B**). The relative confidence interval, the ratio of confidence interval length to the optimal parameter value, is a measure of the resolution of individual parameters, with smaller values indicating better resolution. The full model had 84 parameters, while the simplified model had 36 parameters.

A subset of previously reported flux, metabolite, and protein concentration data for different *E. coli* strains (knockout mutants and their parental strain, BW25113) [16] were used to estimate values and confidence intervals for kinetic parameters used in kinetic rate laws. Non-linear optimization was used to find the optimal set of kinetic parameter values which result in a kinetic model with fluxes and concentrations that are closest to experimental measurements taken from 20 different conditions (different strains and growth rates). For most parameters in the full kinetic model, the relative confidence intervals (ratio of confidence interval length to the optimal parameter value) were large, with more than 80% having a confidence interval that was greater than 100 times the best estimated value (Figure 2A), indicating that we could not be certain of the exact parameter values used in the kinetic rate laws. Similar confidence interval ratios for parameters in these same rate laws were found by Gutenkunst *et al.*[24] when different experimental data was used for estimating parameters (see also Additional file 2: Table S2).

To improve confidence in estimated parameter values, we simplified the kinetic rate laws (Table 1). Optimal parameter values from the full kinetic model were examined, and in many cases, the optimal parameter values resulted in rate laws that were insensitive to changes in metabolite concentrations and/or small changes in parameter values, especially binding coefficients (*K*_*m*_). Though confidence intervals for these parameters were large, it was apparent that some of the parameter values were much smaller or larger than the corresponding metabolite concentrations (parameter values of 10^-6^ or 10^4^ mM versus metabolite concentrations of 10^-3^ to 10^1^ mM). For simple Michaelis-Menten rate laws the binding coefficients could be removed if their values were close to 10^-6^ since they were neglible compared to metabolite concentrations (>10^-3^). Similarly, if the binding coefficients were large (10^4^) compared to the metabolite concentrations (<10^1^) then binding coefficient can be kept and the metabolite concentration ommitted. For these cases, we simplified the functional form of the rate law (Additional file 1: Table S1). As an example, in the full rate law for phosphoglucomutase (PGM), $v=k_{\mathrm{cat}}e_{\mathrm{PGM}}\left(\frac{c_{3\mathrm{pg}}-c_{2\mathrm{pg}}/K_{\mathrm{eq}}}{K_{3\mathrm{pg}}+c_{3\mathrm{pg}}}\right)$ , the optimal value of *K*_3*pg*_ was much smaller than *c*_3*pg*_, so the rate law was effectively $v=k_{\mathrm{cat}}e_{\mathrm{PGM}}\left(\frac{c_{3\mathrm{pg}}-c_{2\mathrm{pg}}/K_{\mathrm{eq}}}{c_{3\mathrm{pg}}}\right)=k_{\mathrm{cat}}e_{\mathrm{PGM}}\left(1-\frac{c_{2\mathrm{pg}}}{c_{3\mathrm{pg}}K_{\mathrm{eq}}}\right)$ since *K*_3*pg*_ + *c*_3*pg*_ ≈ *c*_3*pg*_. For more complicated rate laws, we examined how the flux would change for high (10^1^) and low (10^-3^) metabolite concentrations given the optimal parameter values to identify metabolite concentrations or parameters that could be removed from the simplified rate law. These large and small parameter values lead to poor scaling in the kinetic model during parameter fitting, and the simpler rate laws they suggest would be more efficient to use instead. The parameters that were removed also had large confidence intervals as a result of the kinetic model's insensitivity to those parameters.

**Table 1:** Rate laws used in the simplified model

*v*_*PTS*_	$k_{\mathrm{cat}}e_{\mathrm{PTS}}\frac{c_{\mathrm{pep}}}{c_{\mathrm{pyr}}}$	*v*_*PGI*_	$k_{\mathrm{cat}}e_{\mathrm{PGI}}\left(1-\frac{c_{f6p}}{c_{g6p}K_{\mathrm{eq}}}\right)$
*v*_*PFK*_	*k*_*cat*_*e*_*PFK*_*C*_*atp*_	*v*_*ALDO*_	$\frac{k_{\mathrm{cat}}e_{\mathrm{ALDO}}\left(c_{\mathrm{fdp}}-\frac{c_{\mathrm{gap}}c_{\mathrm{dhap}}}{K_{\mathrm{eq}}}\right)}{K_{\mathrm{fdp}}+C_{\mathrm{fdp}}}$
*v*_*TPI*_	$k_{\mathrm{cat}}e_{\mathrm{TPI}}\left(1-\frac{c_{\mathrm{gap}}}{c_{\mathrm{dhap}}K_{\mathrm{eq}}}\right)$	*v*_*GAPD*_	$k_{\mathrm{cat}}e_{\mathrm{GAPD}}\left(c_{\mathrm{nad}}c_{\mathrm{gap}}-\frac{c_{13\mathrm{dpg}}c_{\mathrm{nadh}}}{K_{\mathrm{eq}}}\right)$
*v*_*PGK*_	$k_{\mathrm{cat}}e_{\mathrm{PGK}}\left(1-\frac{c_{\mathrm{atp}}c_{3\mathrm{pg}}}{c_{\mathrm{adp}}c_{13\mathrm{dpg}}K_{\mathrm{eq}}}\right)$	*v*_*PGM*_	$k_{\mathrm{cat}}e_{\mathrm{PGM}}\left(1-\frac{c_{2\mathrm{pg}}}{c_{3\mathrm{pg}}K_{\mathrm{eq}}}\right)$
*v*_*ENO*_	$k_{\mathrm{cat}}e_{\mathrm{ENO}}\left(c_{2\mathrm{pg}}-\frac{c_{\mathrm{pep}}}{K_{\mathrm{eq}}}\right)$	*v*_*PYK*_	$k_{\mathrm{cat}}e_{\mathrm{PYK}}\frac{c_{\mathrm{adp}}}{c_{\mathrm{atp}}}$
*v*_*PDH*_	$k_{\mathrm{cat}}e_{\mathrm{PDH}}\left(\frac{c_{\mathrm{pyr}}^{4}}{K_{\mathrm{pyr}}+C_{\mathrm{pyr}}^{4}}\right)$	*v*_*PPC*_	$\frac{k_{\mathrm{cat}}e_{\mathrm{PPC}}c_{\mathrm{pep}}\left(1+\frac{c_{\mathrm{fdp}}}{K_{\mathrm{fdp}}}\right)}{K_{\mathrm{pep}}+C_{\mathrm{pep}}}$
*v*_*G*6*PDH*_	*k*_*cat*_*e*_*G*6*PDH*_*c*_*nadp*_*c*_*g*6*p*_	*v*_*GND*_	$\frac{k_{\mathrm{cat}}e_{\mathrm{GND}}c_{\mathrm{nadp}}c_{6\mathrm{pgc}}}{c_{\mathrm{nadph}}c_{\mathrm{atp}}\left(K_{\mathrm{pep}}+C_{\mathrm{pep}}\right)}$
*v*_*RPE*_	$k_{\mathrm{cat}}e_{\mathrm{RPE}}\left(c_{\mathrm{ru}5\mathrm{pD}}-\frac{c_{\mathrm{xu}5\mathrm{pD}}}{K_{\mathrm{eq}}}\right)$	*v*_*RPI*_	$k_{\mathrm{cat}}e_{\mathrm{RPI}}\left(c_{\mathrm{ru}5\mathrm{pD}}-\frac{c_{r5p}}{K_{\mathrm{eq}}}\right)$
*v*_*TKT*1_	$k_{\mathrm{cat}}e_{\mathrm{TKT}1}\left(c_{r5p}c_{\mathrm{xu}5\mathrm{pD}}-\frac{c_{s7p}c_{\mathrm{gap}}}{K_{\mathrm{eq}}}\right)$	*v*_*TALA*_	$k_{\mathrm{cat}}e_{\mathrm{TALA}}\left(c_{\mathrm{gap}}c_{s7p}-\frac{c_{e4p}c_{f6p}}{K_{\mathrm{eq}}}\right)$
*v*_*TKT*2_	$k_{\mathrm{cat}}e_{\mathrm{TKT}2}\left(c_{\mathrm{xu}5\mathrm{pD}}c_{e4p}-\frac{c_{f6p}c_{\mathrm{gap}}}{K_{\mathrm{eq}}}\right)$		

The resulting simplified kinetic model had 36 kinetic parameters (Table 1), compared to 84 parameters in the full rate laws by Chassagnole *et al.*[17]. A WSLS parameter estimation was carried out for the simplified kinetic model, and the optimal parameter and concentration values were found. When the simplified rate laws were used, the value of the WSLS objective function decreased by half, most likely because it was easier to search the feasible space of the simpler kinetic model and find a better solution. The kinetic model simplification process significantly improved the relative confidence intervals for kinetic parameters compared to the full kinetic model (Figure 2). Optimal values and confidence intervals for kinetic parameters can be found in Table 2.

**Table 2:** Estimated parameter values and confidence intervals

	**Parameter**	**Value**	**95% Confidence**	**Units**
PTS	k_cat_	20.7	±9.1	mmol/mg protein/hr
PGI	k_cat_	40.2	±9.5	mmol/mg protein/hr
	K_eq_	1.23	±0.29	Dimensionless
PFK	k_cat_	26	±35	mmol/mg protein/mM/hr
ALDO	k_cat_	3.965	±0.010	mmol/mg protein/mM/hr
	K_eq_	0.18	±0.17	mM
	K_fdp_	0.0074	±0.0036	mM
TPI	k_cat_	10000	±14000	mmol/mg protein/hr
	K_eq_	0.11400	±0.00031	Dimensionless
GAPD	k_cat_	10000	±4100	mmol/mg protein/mM^2^/hr
	K_eq_	1.21	±0.14	Dimensionless
PGM	k_cat_	9995	±40	mmol/mg protein/hr
	K_eq_	0.53570	±0.00063	Dimensionless
PGK	k_cat_	54.3	±2.9	mmol/mg protein/hr
	K_eq_	5512.1	±1.2	Dimensionless
ENO	k_cat_	2	±45	mmol/mg protein/mM/hr
	K_eq_	1.4	±4.1	Dimensionless
PYK	k_cat_	40	±49	mmol/mg protein/hr
PDH	K_cat_	10.4	±4.6	mmol/mg protein/hr
	K_pyr_	0.000020	±0.000029	mM^4^
PPC	k_cat_	2.15	±1.80	mmol/mg protein/hr
	K_fdp_	2.5	±6.5	mM
	K_pep_	0.10	±0.16	mM
G6PDH	k_cat_	859.6	±1.1	mmol/mg protein/mM^2^/hr
GND	k_cat_	18.5	±10.8	mmol*mM/mg protein/hr
	K_6pg_	0.021	±0.012	mM
RPI	k_cat_	549.46	±0.69	mmol/mg protein/mM/hr
	K_eq_	1.40000	±0.00019	Dimensionless
RPE	k_cat_	10000	±13000	mmol/mg protein/mM/hr
	K_eq_	0.4900	±0.0044	Dimensionless
TKT1	k_cat_	10000	±7800	mmol/mg protein/mM^2^/hr
	K_eq_	1.99	±0.012	Dimensionless
TKT2	k_cat_	10000	±5800	mmol/mg protein/mM^2^/hr
	K_eq_	3.500	±0.013	Dimensionless
TALA	k_cat_	10000	±2300	mmol/mg protein/mM^2^/hr
	K_eq_	0.3675	±0.0021	Dimensionless

Binding coefficient parameters have units of *mM*, except for *K*_*pyr*_ in PDH, which has units of *mM*^4^. Equilibrium constants (*K*_*eq*_) are dimensionless, except for ALDO, which has units of *mM*. Values for k_cat_ have units such that fluxes have units of *mmol/gDW/h*. Global upper and lower bounds for binding and enzyme activity were 10^4^ and 10^-6^, respectively. Bounds on equilibrium constants were 0.35*K*_*eq*_^*o*^ and 2.85*K*_*eq*_^*o*^, where *K*_*eq*_^*o*^ is the measured equilibrium constant.

Three types of parameters remained in the simplified kinetic model: enzyme catalytic rate constants (*k*_*cat*_), equilibrium constants (*K*_*eq*_), and binding coefficients (*K*_*m*_). Equilibrium constants had the smallest relative confidence intervals, as changes in these parameters caused very large changes in the kinetic model enzyme and metabolite concentrations. Binding coefficients generally had small relative confidence intervals, as binding coefficients that could not be estimated reliably were eliminated in kinetic model simplification. Enzyme activities had the largest relative confidence intervals, and this was especially true for some reversible reactions. For reversible reactions that are often close to equilibrium (see next section), the catalytic rate constant is much larger than the reaction flux since the mechanistic function, *f*, is close to zero. The enzyme catalytic rate constants cannot be determined for these reactions with precision unless the reaction operates further from equilibrium. Disregarding the reactions that are often near equilibrium, the remaining enzyme activities have relative confidence intervals that are similar to binding and equilibrium constants.

### Evaluation of optimal concentrations

Though parameters for these rate laws have been reported previously in the literature, we found that kinetic parameter values had to differ from these reported *in vitro* and *in vivo* values. When binding coefficient values were fixed to previously-reported *in vitro* or *in vivo* values (reported by Chassagnole *et al.*[17]) in the full kinetic model, no feasible solution could be found to Equations 1b-1e after several thousand nonlinear programming runs from random starting points where *k*_*cat*_ values could vary. Biologically-relevant bounds on the equilibrium constants (*K*_*eq*_) and metabolite or protein concentrations (0.001 to 10 mM and 0.001 to 10 mg protein/gDCW, respectively) in part caused this infeasibility. When bounds were not included in the optimization problem, feasible solutions with large WSLS could be found. In contrast, feasible solutions with small WSLS were found after a few dozen starting points when binding coefficients and *k*_*cat*_ values were all allowed to change simultaneously. This was not surprising, as there have been previous results showing that *in vitro* kinetic parameters are significantly different than their corresponding *in vivo* values [25-27].

**Figure 3:**
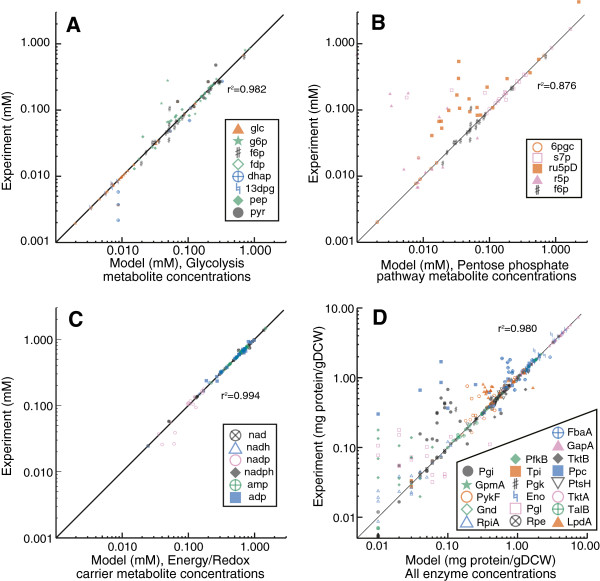
**Model Estimates of All Metabolite and Protein Concentration Measurements.** Comparison between experimental measurements and model estimates of (**A-C**) metabolite and (**D**) protein concentrations for all 20 conditions in the training set. Abbreviations are as follows. glc: glucose, g6p: glucose-6-phosphate, f6p: fructose-6-phosphate, fdp: fructose-1,6-bisphosphate, dhap: dihydroxyacetone phosphate, 13dpg: 1,3-diphosphoglycerate, pep: phosphoenolpyruvate, pyr: pyruvate, 6gpc: 6-phosphogluconate, s7p: sedulose-7-phosphate, ru5pD: rubulose-5-phosphate, nad: nicotinamide adenine dinuceotide (oxidized), nadh: nicotinamide adenine dinuceotide (reduced), nadp: nicotinamide adenine dinuceotide phosphate (oxidized), nadh: nicotinamide adenine dinuceotide phosphate (reduced), GapA: Glyceraldehyde phosphate dehydrogenase, PykF: Pyruvate kinase, Gnd: 6-phosphogluconate dehydrogenase, RpiA: Ribulose 5-phosphate isomerase **A**, TktB: Transketolase II, Ppc: Phosphoenolpyruvate carboxylase, PtsH: Phosphotransferase system, H subunit, PfkB: Phosphofructokinase II, Tpi: Triosephosphate isomerase, Pgk: Phosphoglycerate kinase, Eno: Enolase, Pgl: 6-phosphogluconolactonase, Rpe: Ribulose 5-phosphate epimerase, TktA: Transketolase I, TalB: Transaldolase **B**, LpdA: Lipoamide dehydrogenase (part of pyruvate dehydrogenase complex).

**Figure 4:**
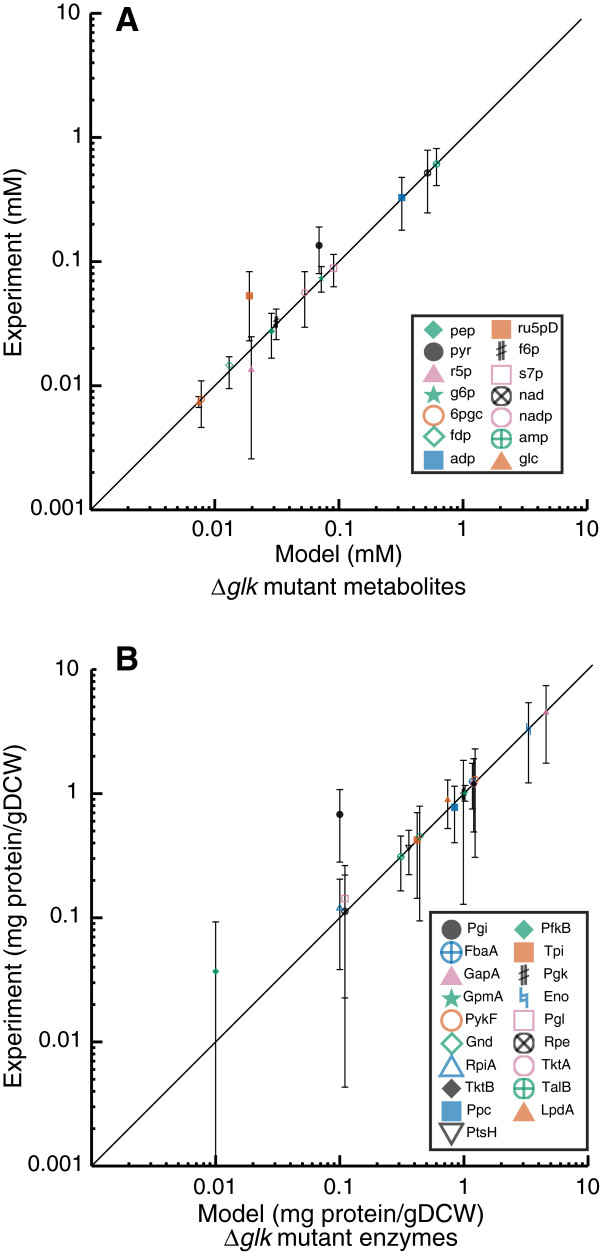
**Comparison Between Estimated and Measured Concentrations with Experimental Errors.** Comparison between experimental measurements and model estimates of metabolite and protein concentrations for a representative condition. Error bars indicate one standard deviation for each measurement. Abbreviations match those in Figure 3.

In addition to comparing kinetic parameters with reported values, we also examined how well the simplified kinetic model fit experimental concentration measurements. Figure 3 shows a comparison between kinetic model predicted concentrations (when the best set of kinetic parameters are used) and experimental observations for metabolite and enzyme concentrations across all 20 conditions that were used to estimate kinetic parameters. Though the predictions did deviate from reported values, for most cases they fell within one standard deviation of the measured values (Figure 4). Considering all 20 conditions, 94.0% of the estimated enzyme concentrations and 89.0% of the kinetic model estimated metabolite concentrations were within one estimated standard deviation of the measured values.

The kinetic model predicted ribose-5-phosphate (r5p) and ribulose-5-phosphate (ru5p) concentrations deviated more from experimental measurements (Figure 3B) as compared to glycolytic and energy/redox carrier metabolites. This was not surprising because the pentose phosphate pathway metabolites had larger experimental variance, and thus were weighted less in WSLS parameter estimation. When all other measurements and kinetic rate laws were excluded from the WSLS problem, the rate laws for RPE and RPI were still difficult to fit to the r5p and ru5p measurements (data not shown), indicating that the proposed rate laws for these two reactions or their associated protein/metabolite measurements may be problematic.

### Kinetic and thermodynamic limitations in central metabolism

**Figure 5:**
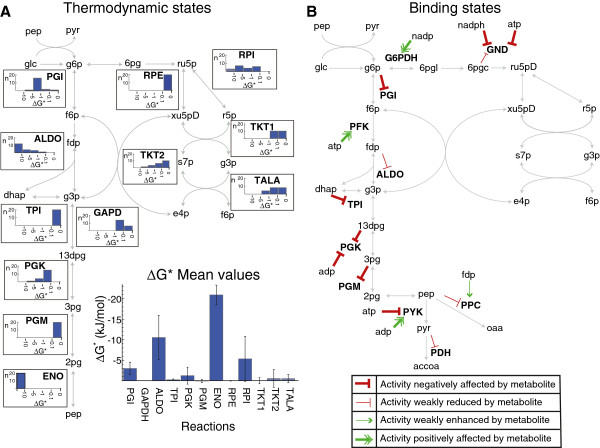
**Thermodynamic Limitations and Metabolite Effects on Enzyme Activities.** Metabolite names are in lowercase and reaction names are in uppercase. (**A**) Histograms of ΔG* in kJ for each reaction across different conditions. Bin height indicates the number of conditions with ΔG* between the two values on the x-axis. Values less than −10 kJ indicate a strong thermodynamic driving force for the reaction, and values near 0 indicate proximity to equilibrium. Mean values and standard deviations for ΔG* for each reaction are also shown at the bottom right. No histograms are shown for irreversible reactions. Abbreviations are as follows. PGI: phosphoglucose isomerase, GAPD: glyceraldehyde phosphate dehydrogenase, ALDO: fructose bisphosphate aldolase, TPI: triosephosphate isomerase, PGK: phosphoglycerate kinase, PGM: phosphoglucomutase, ENO: enolase, RPE: ribulose 5-phosphate epimerase, RPI: ribulose 5-phosphate isomerase, TKT1: transketolase I, TKT2: transketolase 2, TALA: transaldolase (**B**) Apparent secondary effects of metabolite levels in the central metabolism. Inhibition effects are denoted by lines with perpendicular ends. Activation effects are denoted by lines with arrow tips. Intermediate effects (concentration same order of magnitude as binding coefficient) are in yellow. Stronger effects are in red (inhibition) or green (activation).

Since the kinetic parameters included estimates of equilibrium constants (*K*_*eq*_), it was possible to calculate the Gibbs free energy change of reaction in cellular conditions *ΔG*_*j*_ = − *RT* ln(*K*_*eq*,*j*_) and further investigate thermodynamic limitations in central metabolism. Taking the estimates of metabolite concentrations from the best WSLS parameter estimation result, the Gibbs free energy change of reaction in specific conditions was calculated as $\Delta G_{j}^{*}=\Delta G_{j}+\mathrm{RT}\sum_{i}S_{\mathrm{ij}}\ln\left(c_{i}\right)$ (Figure 5A). In many cases, *ΔG*_j_^*^ could not be calculated from the full data set because various concentration measurements were missing. We examined flux control from a thermodynamic perspective because reactions close to equilibrium have lower control on fluxes through the rest of metabolism [28]. It is often assumed that reactions that are far from equilibrium are regulated by microbial cells [7]. Examining the ΔG* values allows us to identify likely metabolic engineering targets without explicitly calculating flux control coefficients.

Across all 20 considered conditions, the mean *ΔG*_ALDO_^*^ and *ΔG*_ENO_^*^ were less than −10 kJ/mol, indicating that fructose-bisphosphate aldolase (ALDO) and enolase (ENO) are often far from equilibrium and among the best targets to control flux through central metabolism [7]. The values for *ΔG*_ALDO_^*^ could be calculated directly from measurements as well, and these were also found to be on the same order of magnitude and far from equilibrium. These results indicate that, although ALDO and ENO are generally considered to be near equilibrium in human erythrocytes [29], this may change depending on the organism and environmental conditions. Other reactions, including pyruvate dehydrogenase (PDH), pyruvate kinase (PYK), phosphofructokinase (PFK), and glucose transport (PTS), are known to be irreversible and thus do not have a *K*_*eq*_ in the kinetic model. These irreversible reactions were observed to have large negative *ΔG*_j_^*^ values in all experimental conditions when *in vitro* measured *ΔG*_*j*_ values in standard conditions were used (data not shown) [21].

Most conditions had similar *ΔG*_j_^*^ values for a given reaction, indicating that the same thermodynamic limitations may arise regardless of the environmental or genetic changes made to the organism. However, five reactions (ribose phosphate isomerase, RPI; transketolase, TKT2; fructose bisphosphate aldolase, ALDO; phosphoglycerate kinase, PGK; and phosphoglucose isomerase, PGI) exhibited different *ΔG*_j_^*^ values for a few conditions, indicating that these five reactions may also control flux through central metabolism under certain conditions. However, the variation in estimated *ΔG*_j_^*^ values for the pentose phosphate reactions (RPI and TKT2) could be due to nosier (and/or missing) metabolite concentration measurements and to larger differences between predicted and measured concentrations for metabolites in this pathway (Figure 3). *ΔG*_j_^*^ variations could also be due to errors in estimated *K*_*eq*_ values, since PGK and PGI both had large *K*_*eq*_ confidence intervals (the confidence intervals for the other three reaction’s *K*_*eq*_ values were small). For the conditions where the *ΔG*_j_^*^ values differed the most we did not observe any consistent patterns in the data that would explain these shifts in *ΔG*_j_^*^ values, such as high or low metabolite concentrations or high or low flux per enzyme concentration values.

Although many binding coefficient parameters were removed during kinetic model simplification, some binding coefficients could be estimated with reasonable confidence intervals. These binding coefficients could then be compared to metabolite concentrations, to identify enzymes whose activity appears to be substantially influenced by metabolite concentrations (Figure 5B). These results can be useful for metabolic engineering applications, as they show the enzyme-metabolite interactions that appear to be biologically-relevant in a variety of growth conditions. For example, inhibition of the glucose transport (PTS) by the pyruvate/phosphoenolpyruvate (*c*_*pyr*_/*c*_*pep*_) ratio was proposed in the rate laws by Chassagnole *et al.*[17] but this inhibition was unimportant and was removed during kinetic model simplification, while the proposed activation of phosphoenolpyruvate carboxylase (PPC) by fructose 1,6-bisphosphate (*C*_*fdp*_) was shown to substantially affect the PPC flux. Because of these kinetic model simplifications, the results clarify which inhibitory and activation effects are biologically-relevant in growing cells.

### Predictions using constraint-based model with kinetic constraints

**Figure 6:**
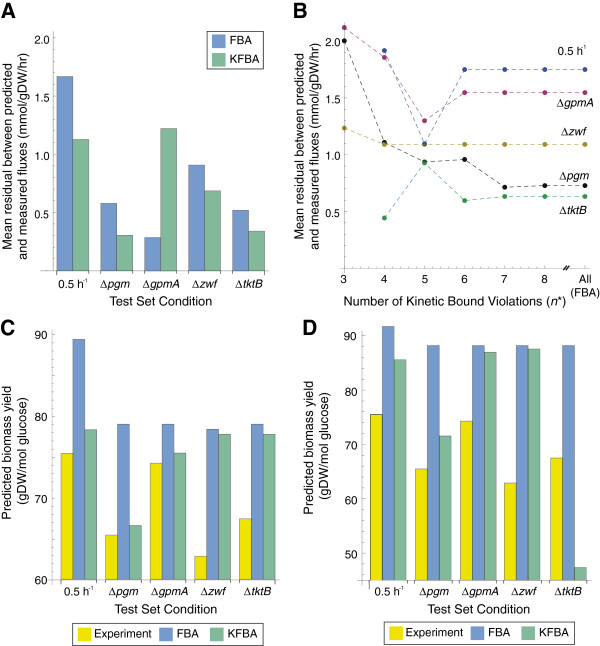
**Kinetic Flux Balance Analysis.** Comparisons between FBA and KFBA predictions for independent test conditions using a central (**A,B**) or genome-scale constraint-based metabolic model (**C,D**). (**A**) Bar height indicates mean residual for each method in the five test conditions using a central metabolic model. Residual was calculated as the squared difference between measurement and FBA or KFBA prediction. (**B**) Bar height indicates the biomass yield from experiments or predictions from FBA or KFBA using a central metabolic model. (**C**) Residuals when using the iJO1366 model in KFBA for different numbers of kinetic flux bound violations (n*). When all flux bounds are ignored the KFBA solution is equivalent to the FBA solution. (**D**) Biomass yields when using the iJO1366 model. KFBA yield predictions (green) correspond to the yield that results when the minimum number of kinetic flux bound violations are allowed.

To evaluate the kinetic model on independent datasets (i.e., those not used to estimate parameters), fluxes in five test conditions were predicted using a constraint-based model with (KFBA) and without (FBA) flux bounds calculated from the kinetic model (Additional file 3). Residuals between experimental and predicted flux values from the constraint-based model were calculated for each test condition, reaction, and algorithm (Figure 6). In addition, biomass yields were predicted using FBA and KFBA by dividing the growth rate by the predicted glucose uptake rate. These flux residuals and biomass yields allowed us to assess how imposing kinetic constraints derived from a kinetic model affect constraint-based model predictions. Overall, using KFBA the flux residuals for the five test conditions were significantly smaller than the flux residuals from FBA (*p=*0.0375 using sign test). In addition, the KFBA predicted biomass yields were closer to experimental values than FBA predicted biomass yields (*p*=0.063 using sign test). Together these results illustrate how kinetic constraints can be used to improve predictions for intracellular fluxes and biomass yield, and demonstrate the applicability of these constraints in conditions outside of the training set.

For the Δ*zwf*, Δ*pgm*, Δ*tktB*, and *D*=0.5 h^-1^ conditions, the mean residual from KFBA was smaller than the mean residual from FBA (Figure 6A). Uptake and secretion fluxes were also better predicted for these conditions. The Δ*pgm* mutant has a growth defect (biomass yields are reduced by ~12% [16]), needing larger glucose uptake and carbon dioxide secretion rates when compared to the parental strain at the same growth rate. However, FBA predicts the same biomass yields for the Δ*pgm* mutant as the parental strain at a growth rate of 0.2 h^-1^ and has 16.5% error and 27.7% error in the predicted glucose uptake and carbon dioxide production rates, respectively. The KFBA algorithm correctly predicts a lower Δ*pgm* mutant biomass yield than the parental strain (Figure 6B), with just a 1% error and 3.2% error in the glucose uptake and carbon dioxide production rates, respectively. In this KFBA solution, PDH, PPC, and TKT2 reactions are all at their lower kinetic bounds, and carbon is forced through glycolysis and the pentose phosphate pathway that is not ultimately incorporated into biomass, and is instead secreted as carbon dioxide.

Not all predictions were improved by using the kinetic constraints. The residuals for the intracellular fluxes predicted by KFBA for the Δ*gpmA* mutant were greater than those predicted by FBA. In this case, the kinetic bounds associated with the PTS and PDH reactions caused KFBA to incorrectly predict fluxes through glycolysis, contributing to the large mean residual for KFBA. Removing the PTS and PDH bounds reduced the mean residual to 0.39, similar to FBA.

We also repeated the FBA and KFBA analysis using the iJO1366 genome-scale constraint-based model. The iJO1366 model contains more reactions in and around central metabolism. As a result, we found that fewer kinetic constraints need to be violated, as compared to the central model, and that pathways were predicted to be used that are not normally operational under glucose aerobic conditions (e.g., non-PTS glucose transport, glucose dehydrogenase, gluconate kinase, isocitrate lyase and xylose isomerase). We further evaluated how the KFBA solutions changed as the number of allowable kinetic flux bound violations (n*) increased (Figure 6C). These results show that there is a tradeoff between maximizing agreement with the kinetic flux bounds and not activating additional pathways (not included in the central metabolic model), which causes poorer agreement with experimental flux measurements. As observed with the central metabolic model, the biomass yields were predicted better when kinetic constraints were imposed (Figure 6D). To improve KFBA predictions of central metabolic fluxes, kinetic constraints for additional pathways (noted above) in the iJO1366 model need to be included.

## Conclusions

Constraint-based modeling uses mass balances, flux capacities, reaction directionalities, and other relevant physical constraints to predict fluxes through metabolism. Although transcriptional regulatory and thermodynamic constraints have been integrated into this modeling approach, detailed kinetic constraints have not been extensively formulated, parameterized, and used in constraint-based models. Since kinetic constraints are often omitted from constraint-based models, some predicted flux distributions may not be achievable using native enzymes or protein levels. Incorporation of kinetic constraints into constraint-based allows multi-omic datasets to be used to find kinetic limitations on metabolic fluxes and suggests enzymes to target for improving cell behavior. For example, the Δ*pgm* mutant is predicted to have lower biomass yields due to kinetic limitations, and the KFBA model suggests that decreasing PDH, PPC, and TKT2 levels would improve biomass yields for this mutant. One challenge with developing such kinetically-constrained models is finding kinetic parameter values that are consistent with experimental measurements.

In this study, a WSLS parameter estimation problem using multi-omic experimental data from a study by Ishii *et al.*[16] was formulated and solved. The parameter estimation results suggested changes to functional forms of rate laws, which were implemented to produce a simplified kinetic model. The simpler kinetic model is an improvement over the more detailed model because the parameters are better-resolved and the model could be solved more efficiently with a better fit to experimental data. Each of the retained metabolite-enzyme binding coefficient parameters were associated with measured metabolite concentrations, and more binding parameters could be retained in the future by measuring concentrations for more chemical species. Overall, the kinetic parameters we estimated could fit the kinetic model to 92.7% of the 720 measured metabolite and protein concentration measurements within one standard deviation across 20 different experiments.

The thermodynamic predictions about distance from equilibrium can also be used for metabolic engineering applications [7]. In this case, reactions that are far from equilibrium may be limited by kinetic regulation or enzyme levels, preventing them from reaching equilibrium. Here, we identified reactions that were far from equilibrium during growth in most conditions, which represent viable candidates for modifications to control flux through metabolism. The results of thermodynamic analysis from this study are consistent with other thermodynamic analysis of microbes. In a study by Kümmel *et al.*[7], it was found that the ALDO reaction in *E. coli* is not near equilibrium under physiological conditions. Klimacek *et al.*[30] found that the ALDO and ENO reactions are far from equilibrium in wild type and engineered *Saccharomyces cerevisiae* strains. Our results combined with these earlier studies indicate that *E. coli* and *S. cerevisiae* must use significantly different strategies for regulation of glycolysis than human erythrocytes, which instead closely regulate entry into and exit from glycolysis, and have reactions near equilibrium for all intermediate steps [29]. Enzymes were also identified where metabolite concentrations had significant binding, saturation, and allosteric regulatory effects. The conclusions about thermodynamics and enzyme binding are based on a simultaneous analysis of metabolomic, proteomic, and fluxomic data.

When kinetic constraints were imposed on a central metabolic constraint-based model the flux and biomass yield predictions were more accurately predicted by KFBA than FBA. Imposition of kinetic constraints in a genome-scale model provided mixed results, with more accurate biomass yields but worse overall flux residuals after incorporation of kinetic constraints. Since additional pathways are utilized in iJO1366 to match more kinetic flux bounds, the application of more kinetic constraints could improve predictions in more comprehensive models. The confidence intervals for kinetic parameters allowed reasonable flux ranges to be estimated from metabolite and enzyme concentration data. For the five test conditions that were evaluated, a median of 16 (out of 22) kinetic flux bounds were feasible. We considered potential errors in estimated parameter values to calculate the kinetic flux bounds, but errors in metabolite and protein concentration measurements could also be considered. The KFBA method used in this work could be applied to other reported rate laws, kinetic parameters, and concentration data, provided reasonable flux ranges can be calculated. However, identifying reasonable flux ranges may require parameter confidence intervals, which are not always available.

The presented approach to parameterize kinetic rate laws using *in vivo* data is general and can be applied to multi-omics data from other microbes. We chose published rate laws for a starting point for our kinetic model, but we note that these rate laws are almost entirely based on mass-action kinetics and Michaelis-Menten type inhibition. These rate laws were sufficient for our system, and we suggest the use of similar expressions for pathways where rate laws have not been proposed. The methods for imposing kinetic constraints in constraint-based models are also general, and can be used with rate laws with *in vitro* or *in vivo* determined parameters. Methods to parameterize and use kinetic rate laws in constraint-based models will benefit from more global and precise metabolomics and proteomics methods. Future work will involve including rate laws for other metabolic pathways (such as the citric acid cycle, fermentative and respiratory pathways) and estimating their *in vivo* kinetic parameters. Overall, this work illustrates how kinetic constraints can be used to improve predictions for intracellular fluxes and biomass yield and identify potential metabolic limitations through the integrated analysis of multi-omics datasets.

## Competing interests

The authors declare that they have no competing interests.

## Author’s contributions

CC implemented the models and approach, performed the analysis, analyzed the data, and drafted the manuscript. JLR conceived of the study, participated in its design and coordination, and helped to analyze the data and draft the manuscript. All authors read and approved the final manuscript.

## Supplementary Material

Additional file 1 **Contains Table S1 – Rate laws used in the full and simplified models.** Rate laws in the full model (middle column) and simplified model (right column). Each rate law in the simplified model was deduced directly from the corresponding rate law and optimal parameter values in the full model. Parameters shown in bold face are those that had relative confidence intervals exceeding 100. Click here for file

Additional file 2 **Contains Table S2 - Estimated parameter values and confidence intervals.** Binding coefficient parameters have units of *mM*, except for K_pyr_ in PDH, which has units of *mM*^4^. Equilibrium constants (K_eq_) are dimensionless, except for ALDO, which has units of *mM*. Values for k_cat_ have units such that fluxes have units of *mmol/gDW/h*. Units for k_cat_ may differ between full and simplified models. Click here for file

Additional file 3 Contains the Kinetic Model in SBML format. Click here for file

## References

[B1] Price ND Reed JL Palsson BO Genome-scale models of microbial cells: evaluating the consequences of constraints Nat Rev Microbiol 2004 2 11 886 897 10.1038/nrmicro1023 15494745

[B2] Orth JD Thiele I Palsson BO What is flux balance analysis? Nat Biotechnol 2010 28 3 245 248 10.1038/nbt.1614 20212490 PMC3108565

[B3] Schuetz R Kuepfer L Sauer U Systematic evaluation of objective functions for predicting intracellular fluxes in Escherichia coli Mol Syst Biol 2007 3 119 17625511 10.1038/msb4100162 PMC1949037

[B4] Covert MW Knight EM Reed JL Herrgard MJ Palsson BO Integrating high-throughput and computational data elucidates bacterial networks Nature 2004 429 6987 92 96 10.1038/nature02456 15129285

[B5] Chandrasekaran S Price ND Probabilistic integrative modeling of genome-scale metabolic and regulatory networks in Escherichia coli and Mycobacterium tuberculosis Proc Natl Acad Sci USA 2010 107 41 17845 17850 10.1073/pnas.1005139107 20876091 PMC2955152

[B6] Shlomi T Eisenberg Y Sharan R Ruppin E A genome-scale computational study of the interplay between transcriptional regulation and metabolism Mol Syst Biol 2007 3 101 17437026 10.1038/msb4100141 PMC1865583

[B7] Kümmel A Panke S Heinemann M Putative regulatory sites unraveled by network-embedded thermodynamic analysis of metabolome data Mol Syst Biol 2006 2 2006 0034 16788595 10.1038/msb4100074 PMC1681506

[B8] Henry CS Broadbelt LJ Hatzimanikatis V Thermodynamics-based metabolic flux analysis Biophys J 2007 92 5 1792 1805 10.1529/biophysj.106.093138 17172310 PMC1796839

[B9] Hoppe A Hoffmann S Holzhutter HG Including metabolite concentrations into flux balance analysis: thermodynamic realizability as a constraint on flux distributions in metabolic networks BMC Syst Biol 2007 1 23 10.1186/1752-0509-1-23 17543097 PMC1903363

[B10] Henry CS Jankowski MD Broadbelt LJ Hatzimanikatis V Genome-scale thermodynamic analysis of Escherichia coli metabolism Biophys J 2006 90 4 1453 1461 10.1529/biophysj.105.071720 16299075 PMC1367295

[B11] Beg QK Vazquez A Ernst J de Menezes MA Bar-Joseph Z Barabasi AL Oltvai ZN Intracellular crowding defines the mode and sequence of substrate uptake by Escherichia coli and constrains its metabolic activity Proc Natl Acad Sci USA 2007 104 31 12663 12668 10.1073/pnas.0609845104 17652176 PMC1937523

[B12] Yizhak K Benyamini T Liebermeister W Ruppin E Shlomi T Integrating quantitative proteomics and metabolomics with a genome-scale metabolic network model Bioinformatics 2010 26 12 i255 260 10.1093/bioinformatics/btq183 20529914 PMC2881368

[B13] Hanly TJ Henson MA Dynamic flux balance modeling of microbial co-cultures for efficient batch fermentation of glucose and xylose mixtures Biotechnol Bioeng 2010 108 2 376 385 20882517 10.1002/bit.22954

[B14] Mahadevan R Edwards JS Doyle FJ 3rd Dynamic flux balance analysis of diauxic growth in Escherichia coli Biophys J 2002 83 3 1331 1340 10.1016/S0006-3495(02)73903-9 12202358 PMC1302231

[B15] Zhuang K Izallalen M Mouser P Richter H Risso C Mahadevan R Lovley DR Genome-scale dynamic modeling of the competition between Rhodoferax and Geobacter in anoxic subsurface environments ISME J 2010 5 2 305 316 20668487 10.1038/ismej.2010.117 PMC3105697

[B16] Ishii N Nakahigashi K Baba T Robert M Soga T Kanai A Hirasawa T Naba M Hirai K Hoque A Multiple high-throughput analyses monitor the response of E. coli to perturbations Science 2007 316 5824 593 597 10.1126/science.1132067 17379776

[B17] Chassagnole C Noisommit-Rizzi N Schmid JW Mauch K Reuss M Dynamic modeling of the central carbon metabolism of Escherichia coli Biotechnol Bioeng 2002 79 1 53 73 10.1002/bit.10288 17590932

[B18] Palsson B Systems biology: properties of reconstructed networks 2006 Cambridge; New York: Cambridge University Press

[B19] Orth JD Conrad TM Na J Lerman JA Nam H Feist AM Palsson BO A comprehensive genome-scale reconstruction of Escherichia coli metabolism–2011 Mol Syst Biol 2011 7 535 21988831 10.1038/msb.2011.65 PMC3261703

[B20] Chapman AG Fall L Atkinson DE Adenylate energy charge in Escherichia coli during growth and starvation J Bacteriol 1971 108 3 1072 1086 4333317 10.1128/jb.108.3.1072-1086.1971 PMC247190

[B21] Goldberg RN Tewari YB Bhat TN Thermodynamics of enzyme-catalyzed reactions–a database for quantitative biochemistry Bioinformatics 2004 20 16 2874 2877 10.1093/bioinformatics/bth314 15145806

[B22] Antoniewicz MR Kelleher JK Stephanopoulos G Determination of confidence intervals of metabolic fluxes estimated from stable isotope measurements Metab Eng 2006 8 4 324 337 10.1016/j.ymben.2006.01.004 16631402

[B23] Lewis NE Hixson KK Conrad TM Lerman JA Charusanti P Polpitiya AD Adkins JN Schramm G Purvine SO Lopez-Ferrer D Omic data from evolved E. coli are consistent with computed optimal growth from genome-scale models Mol Syst Biol 2010 6 390 20664636 10.1038/msb.2010.47 PMC2925526

[B24] Gutenkunst RN Waterfall JJ Casey FP Brown KS Myers CR Sethna JP Universally sloppy parameter sensitivities in systems biology models PLoS Comput Biol 2007 3 10 1871 1878 17922568 10.1371/journal.pcbi.0030189 PMC2000971

[B25] Van Eunen K Kiewiet JA Westerhoff HV Bakker BM Testing biochemistry revisited: how in vivo metabolism can be understood from in vitro enzyme kinetics PLoS Comput Biol 2012 8 4 e1002483 10.1371/journal.pcbi.1002483 22570597 PMC3343101

[B26] Huang X Holden HM Raushel FM Channeling of substrates and intermediates in enzyme-catalyzed reactions Annu Rev Biochem 2001 70 149 180 10.1146/annurev.biochem.70.1.149 11395405

[B27] Ellis RJ Macromolecular crowding: obvious but underappreciated Trends Biochem Sci 2001 26 10 597 604 10.1016/S0968-0004(01)01938-7 11590012

[B28] Wang L Birol I Hatzimanikatis V Metabolic control analysis under uncertainty: framework development and case studies Biophys J 2004 87 6 3750 3763 10.1529/biophysj.104.048090 15465856 PMC1304888

[B29] Garrett R Grisham CM Biochemistry 1995 Fort Worth: Saunders College Pub

[B30] Klimacek M Krahulec S Sauer U Nidetzky B Limitations in xylose-fermenting Saccharomyces cerevisiae, made evident through comprehensive metabolite profiling and thermodynamic analysis Appl Environ Microbiol 2010 76 22 7566 7574 10.1128/AEM.01787-10 20889786 PMC2976174

