# Supplementary material for: Mechanistic analysis of multi-omics datasets to generate kinetic parameters for constraint-based metabolic models
Source: BMC Bioinformatics. 2013 Jan 30;14:32. doi: 10.1186/1471-2105-14-32 (PMC3571921; doi:10.1186/1471-2105-14-32)
Supplement: Additional file 1 — Contains Table S1 – Rate laws used in the full and simplified models. Rate laws in the full model (middle column) and simplified model (right column). Each rate law in the simplified model was deduced directly from the corresponding rate law and optimal parameter values in the full model. Parameters shown in bold face are those that had relative confidence intervals exceeding 100. [file 1471-2105-14-32-S1.pdf]

Supplementary Table 1: Rate laws in the full model (middle column) and simplified model (right column). Each rate law in the simplified model was deduced directly from the corresponding rate law and optimal parameter values in the full model. Parameters shown in bold face are those that had relative confidence intervals exceeding 100.

Flux	Full Rate Law (Chassagnole *et al.*, 2002)	Simplified Rate Law
v_{PTS}	$k_{cat}e_{PTS}\dfrac{C_{glc}\dfrac{C_{pep}}{C_{pyr}}}{\left(\boldsymbol{K_{a1}}+\boldsymbol{K_{a2}}\dfrac{C_{pep}}{C_{pyr}}+\boldsymbol{K_{a3}}+C_{glc}\dfrac{C_{pep}}{C_{pyr}}\right)\left(1+\dfrac{C_{g6p}^{n}}{\boldsymbol{K_{g6p}}}\right)}$	$k_{cat}e_{PTS}\dfrac{C_{pep}}{C_{pyr}}$
v_{PGI}	$\boldsymbol{k_{cat}}e_{PGI}\dfrac{\left(C_{g6p}-\dfrac{C_{f6p}}{K_{eq}}\right)}{\boldsymbol{K_{g6p}}\left(1+\dfrac{C_{f6p}}{\boldsymbol{K_{f6p}}\left(1+\dfrac{C_{6pgc}}{\boldsymbol{K_{f6p,6pgcinh}}}\right)}+\dfrac{C_{6pc}}{\boldsymbol{K_{g6p,6pgcinh}}}\right)+C_{g6p}}$	$k_{cat}e_{PGI}\left(1-\dfrac{C_{f6p}}{C_{g6p}K_{eq}}\right)$
v_{PFK}	$\boldsymbol{k_{cat}}e_{PFK}\dfrac{C_{atp}C_{f6p}}{\left(C_{atp}+\boldsymbol{K_{atps}}\left(1+\dfrac{C_{adp}}{\boldsymbol{K_{adpc}}}\right)\right)\left(C_{f6p}+\boldsymbol{K_{f6ps}}\dfrac{A}{B}\right)\left(1+\dfrac{L}{\left(1+\dfrac{C_{f6p}}{\boldsymbol{K_{f6ps}}}\dfrac{A}{B}\right)^{n}}\right)}$ with $A=1+\frac{C_{pep}}{\boldsymbol{K_{pep}}}+\frac{C_{adp}}{\boldsymbol{K_{adpb}}}+\frac{C_{amp}}{\boldsymbol{K_{ampb}}}$ and $B=1+\frac{C_{adp}}{\boldsymbol{K_{adpa}}}+\frac{C_{amp}}{\boldsymbol{K_{ampa}}}$	$k_{cat}e_{PFK}C_{atp}$
v_{ALDO}	$k_{cat}e_{ALDO}\dfrac{\left(C_{fdp}-\dfrac{C_{gap}C_{dhap}}{K_{eq}}\right)}{K_{fdp}+C_{fdp}+\boldsymbol{K_{gap}}\dfrac{C_{dhap}}{K_{eq}\boldsymbol{V_{blf}}}+\boldsymbol{K_{dhap}}\dfrac{C_{gap}}{K_{eq}\boldsymbol{V_{blf}}}+\dfrac{C_{fdp}C_{gap}}{\boldsymbol{K_{gap,inh}}}+\dfrac{C_{dhap}C_{gap}}{K_{eq}\boldsymbol{V_{blf}}}}$	$\dfrac{k_{cat}e_{FBA}\left(C_{fdp}-\dfrac{C_{gap}C_{dhap}}{K_{eq}}\right)}{K_{fdp}+C_{fdp}}$
v_{TPI}	$\boldsymbol{k_{cat}}e_{TPI}\dfrac{\left(C_{dhap}-\dfrac{C_{gap}}{\boldsymbol{K_{eq}}}\right)}{\boldsymbol{K_{dhap}}\left(1+\dfrac{C_{gap}}{\boldsymbol{K_{gap}}}\right)+C_{dhap}}$	$k_{cat}e_{TPI}\left(1-\dfrac{C_{gap}}{C_{dhap}K_{eq}}\right)$
v_{GAPD}	$\boldsymbol{k_{cat}}e_{GAPD}\dfrac{\left(C_{gap}C_{nad}-\dfrac{C_{13dpg}C_{nadh}}{\boldsymbol{K_{eq}}}\right)}{\left(\boldsymbol{K_{gap}}\left(1+\dfrac{C_{13dpg}}{\boldsymbol{K_{13dpg}}}\right)+C_{gap}\right)\left(\boldsymbol{K_{nad}}\left(1+\dfrac{C_{nadh}}{\boldsymbol{K_{nadh}}}\right)+C_{nad}\right)}$	$k_{cat}e_{GAPD}\left(C_{nad}C_{gap}-\dfrac{C_{13dpg}C_{nadh}}{K_{eq}}\right)$
v_{PGK}	$\boldsymbol{k_{cat}}e_{PGK}\dfrac{\left(C_{adp}C_{13dpg}-\dfrac{C_{atp}C_{3pg}}{\boldsymbol{K_{eq}}}\right)}{\left(\boldsymbol{K_{adp}}\left(1+\dfrac{C_{atp}}{\boldsymbol{K_{atp}}}\right)+C_{adp}\right)\left(\boldsymbol{K_{13dpg}}\left(1+\dfrac{C_{3pg}}{\boldsymbol{K_{3pg}}}\right)+C_{13dpg}\right)}$	$k_{cat}e_{PGK}\left(1-\dfrac{C_{atp}C_{3pg}}{C_{atp}C_{13dpg}K_{eq}}\right)$
v_{PGM}	$k_{cat}e_{PGM}\dfrac{\left(C_{3pg}-\dfrac{C_{2pg}}{K_{eq}}\right)}{\boldsymbol{K_{3pg}}\left(1+\dfrac{C_{2pg}}{\boldsymbol{K_{2pg}}}\right)+C_{3pg}}$	$k_{cat}e_{PGM}\left(1-\dfrac{C_{2pg}}{C_{3pg}K_{eq}}\right)$
v_{ENO}	$\boldsymbol{k_{cat}}e_{ENO}\dfrac{\left(C_{2pg}-\dfrac{C_{pep}}{\boldsymbol{K_{eq}}}\right)}{\boldsymbol{K_{2pg}}\left(1+\dfrac{C_{pep}}{\boldsymbol{K_{pep}}}\right)+C_{2pg}}$	$k_{cat}e_{ENO}\left(C_{2pg}-\dfrac{C_{pep}}{K_{eq}}\right)$

v_{PYK}	$k_{cat}e_{PYK}\dfrac{C_{pep}\left(\dfrac{C_{pep}}{\boldsymbol{K_{pep}}}+1\right)^{n-1}C_{adp}}{\boldsymbol{K_{pep}}\left(L\left(\dfrac{1+\dfrac{C_{atp}}{\boldsymbol{K_{atp}}}}{1+\dfrac{C_{fdp}}{\boldsymbol{K_{fdp}}}+\dfrac{C_{amp}}{\boldsymbol{K_{amp}}}}\right)^{n}+\left(1+\dfrac{C_{pep}}{\boldsymbol{K_{pep}}}\right)^{n}\right)\left(C_{adp}+\boldsymbol{K_{adp}}\right)}$	$k_{cat}e_{PYK}\dfrac{C_{adp}}{C_{atp}}$
v_{PDH}	$k_{cat}e_{PDH}\dfrac{C_{pyr}^{n}}{\boldsymbol{K_{pyr}}+C_{pyr}^{4}}$	$k_{cat}e_{PDH}\dfrac{C_{pyr}^{4}}{K_{pyr}+C_{pyr}^{4}}$
v_{PPC}	$\boldsymbol{k_{cat}}e_{PPC}\dfrac{C_{pep}\left(1+\dfrac{C_{fdp}}{\boldsymbol{K_{fdp}}}\right)}{K_{pep}+C_{pep}}$	$k_{cat}e_{PPC}\dfrac{C_{pep}\left(1+\dfrac{C_{fdp}}{K_{fpd}}\right)}{K_{pep}+C_{pep}}$
v_{G6PDH}	$\boldsymbol{k_{cat}}e_{G6PDH}\dfrac{C_{g6p}C_{napd}}{\left(\boldsymbol{K_{g6p}}+C_{g6p}\right)\left(1+\dfrac{C_{nadph}}{\boldsymbol{K_{nadph,g6pinh}}}\right)\left(\boldsymbol{K_{nadp}}\left(1+\dfrac{C_{nadph}}{\boldsymbol{K_{nadph,nadhinh}}}\right)\right)}$	$k_{cat}e_{G6PDH}C_{nadp}C_{g6p}$
v_{GND}	$\boldsymbol{k_{cat}}e_{GND}\dfrac{C_{6pgc}C_{nadp}}{\left(K_{6pgc}+C_{6pgc}\right)\left(\boldsymbol{K_{nadp}}\left(1+\dfrac{C_{nadph}}{\boldsymbol{K_{nadphinh}}}\right)\left(1+\dfrac{C_{atp}}{\boldsymbol{K_{atpinh}}}\right)+C_{nadp}\right)}$	$\dfrac{k_{cat}e_{GND}C_{nadp}C_{6pgc}}{C_{nadph}C_{atp}\left(K_{6pgc}+C_{6pgc}\right)}$
v_{RPE}	$k_{cat}e_{RPE}\left(C_{ru5pD}-\dfrac{C_{xu5pD}}{K_{eq}}\right)$	$k_{cat}e_{RPE}\left(C_{ru5pD}-\dfrac{C_{xu5pD}}{K_{eq}}\right)$
v_{RPI}	$k_{cat}e_{RPI}\left(C_{ru5pD}-\dfrac{C_{r5p}}{K_{eq}}\right)$	$k_{cat}e_{RPI}\left(C_{ru5pD}-\dfrac{C_{r5p}}{K_{eq}}\right)$
v_{TKT1}	$k_{cat}e_{TKT1}\left(C_{r5p}C_{xu5pD}-\dfrac{C_{s7p}C_{gap}}{K_{eq}}\right)$	$k_{cat}e_{TKT1}\left(C_{r5p}C_{xu5pD}-\dfrac{C_{s7p}C_{gap}}{K_{eq}}\right)$
v_{TKT2}	$k_{cat}e_{TKT2}\left(C_{xu5pD}C_{e4p}-\dfrac{C_{f6p}C_{gap}}{K_{eq}}\right)$	$k_{cat}e_{TKT2}\left(C_{xu5pD}C_{e4p}-\dfrac{C_{f6p}C_{gap}}{K_{eq}}\right)$
v_{TALA}	$\boldsymbol{k_{cat}}e_{TALA}\left(C_{gap}C_{s7p}-\dfrac{C_{e4p}C_{f6p}}{K_{eq}}\right)$	$k_{cat}e_{TALA}\left(C_{gap}C_{s7p}-\dfrac{C_{e4p}C_{f6p}}{K_{eq}}\right)$
