# Supplementary material for: Mechanistic analysis of multi-omics datasets to generate kinetic parameters for constraint-based metabolic models
Source: BMC Bioinformatics. 2013 Jan 30;14:32. doi: 10.1186/1471-2105-14-32 (PMC3571921; doi:10.1186/1471-2105-14-32)
Supplement: Additional file 2 — Contains Table S2 - Estimated parameter values and confidence intervals. Binding coefficient parameters have units of mM, except for Kpyr in PDH, which has units of mM4. Equilibrium constants (Keq) are dimensionless, except for ALDO, which has units of mM. Values for kcat have units such that fluxes have units of mmol/gDW/h. Units for kcat may differ between full and simplified models. [file 1471-2105-14-32-S2.pdf]

Supplementary Table 2 - Estimated parameter values and confidence intervals. Binding coefficient parameters have units of *mM*, except for K_{pyr} in PDH, which has units of mM^4. Equilibrium constants (K_{eq}) are dimensionless, except for ALDO, which has units of *mM*. Values for k_{cat} have units such that fluxes have units of *mmol/gDW/h*. Units for k_{cat} may differ between full and simplified models.

		Full Model		Simplified Model	
	Parameter	Value	95% Confidence	Value	95% Confidence
PTS	k_{cat}	18	∞	20.7	±9.1
	K_{a1}	0.00001	∞		
	K_{a2}	0.0022	∞		
	K_{a3}	0.012	∞		
	K_{g6p}	0.35	∞		
PGI	k_{cat}	1000000	∞	40.2	±9.5
	K_{eq}	0.411	∞	1.23	±0.29
	K_{g6p}	0.432	∞		
	K_{f6p}	3000	∞		
	$K_{f6p,6pg,inh}$	5.26	∞		
	$K_{g6p,6pg,inh}$	0.00001	∞		
PFK	k_{cat}	1000000	∞	26	±35
	$K_{atp,s}$	62367	∞		
	$K_{adp,c}$	1000000	∞		
	$K_{f6p,s}$	0.00001	∞		
	K_{pep}	1000000	∞		
	$K_{adp,b}$	1000000	∞		
	$K_{amp,b}$	1000000	∞		
	$K_{adp,a}$	2200	∞		
	$K_{amp,a}$	0.00001	∞		
	L	4000000	∞		
FBA	k_{cat}	4.5	±2.8	3.965	±0.010
	K_{eq}	0.18	±0.26	0.18	±0.17
	K_{fdp}	0.0065	±0.0067	0.0074	±0.0036
	K_{gap}	0.00001	∞		
	K_{blf}	0.52	∞		
	K_{dhap}	0.00001	∞		
	$K_{gah,inh}$	1000000	∞		
TPI	k_{cat}	1000000	∞	10000	±14000
	K_{eq}	0.114	∞	0.11400	±0.00031
	K_{dhap}	0.00001	∞		
	K_{gap}	14	∞		
GAPDH	k_{cat}	1000000	∞	10000	±4100
	K_{eq}	1.6	∞	1.21	±0.14
	K_{gap}	0.00001	∞		
	K_{13dpg}	20000	∞		
	K_{nad}	0.030	∞		
	K_{nadh}	0.00001	∞		
PGK	k_{cat}	56	∞	54.3	±2.9
	K_{eq}	5500	∞	5512.1	±1.2
	K_{adp}	0.26	∞		
	K_{atp}	1000000	∞		
	K_{13dpg}	0.0032	∞		
	K_{3pg}	1.4	∞		
PGM	k_{cat}	1000000	±2300	9995	±40
	K_{eq}	0.5358	±0.0017	0.53570	±0.00063
	K_{3pg}	640000	∞		

	K_{2pg}	1000000	∞		
ENO	k_{cat}	1000000	∞	1.59	±0.02
	K_{eq}	19.18	∞	19.2	±0.25
	K_{2pg}	1300	∞		
	K_{pep}	460000	∞		
PYK	k_{cat}	37	±23	40	±49
	K_{pep}	0.00001	∞		
	L	1000	∞		
	K_{atp}	0.086	∞		
	K_{fdp}	12	∞		
	K_{amp}	29	∞		
	K_{adp}	0.00001	∞		
PDH	k_{cat}	18.76	∞	10.4	±4.6
	K_{pyr}	0.0015	∞	0.000020	±0.000029
PPC	k_{cat}	2.1091	±0.0067	2.15	±1.80
	K_{fdp}	2.4	±1.5	2.5	±6.5
	K_{pep}	0.0972	±0.0013	0.10	±0.16
RPI	k_{cat}	102	±146	549.46	±0.69
	K_{eq}	2.8	±3.0	1.40000	±0.00019
RPE	k_{cat}	94	±53	10000	±13000
	K_{eq}	4.0	±2.3	0.4900	±0.0044
GND	k_{cat}	1000000	∞	18.5	±10.8
	K_{6pg}	224.93	±0.47	0.021	±0.012
	K_{nadp}	6.1	∞		
	$K_{nadph,inh}$	1.5	∞		
	$K_{atp,inh}$	2.4	∞		
G6PDH	k_{cat}	760000	∞	859.6	±1.1
	K_{g6p}	31	±8185		
	$K_{nadph,g6p,inh}$	1.8	±23508		
	K_{nadp}	3.4	±26739		
	$K_{nadph,nadp,inh}$	1.8	±7428		
TKT1	k_{cat}	15.1	±7.9	10000	±7800
	K_{eq}	1.9	±2.8	1.99	±0.012
TKT2	k_{cat}	5.1	∞	10000	±5800
	K_{eq}	3.5	±9.0	3.500	±0.013
TALA	k_{cat}	12000	∞	10000	±2300
	K_{eq}	0.37	±0.58	0.3675	±0.0021
